# Chest pain with elevated ST segment in lead V1: a case report

**DOI:** 10.3389/fcvm.2026.1731030

**Published:** 2026-03-06

**Authors:** Zhongyong He, Xiaobo Pu, Lijun Zeng

**Affiliations:** 1 Department of Cardiology, Mianyang People’s Hospital, Mianyang, China; 2 Department of Cardiology, West China Hospital, Sichuan University, Chengdu, China; 3 Department of General Internal Medicine, West China Second University Hospital, Sichuan University, Chengdu, China

**Keywords:** differential diagnoses, electrocardiogram, isolated right ventricular infarction, non-dominant right coronary artery, preload optimization

## Abstract

Isolated right ventricular infarction (RVI) is an underrecognized clinical entity that often mimics anteroseptal myocardial infarction due to its characteristic ST-segment elevation (STE) in precordial leads. We report the case of a 55-year-old man who presented with acute chest pain and STE in leads V1 and V2. Despite typical anteroseptal infarction patterns, the electrocardiogram (ECG) revealed decremental STE (V1 > V2), concomitant STE in lead III, and reciprocal ST depression in lateral leads. Coronary angiography confirmed proximal occlusion of the non-dominant right coronary artery. This case highlights the diagnostic challenges of isolated RVI and underscores key ECG features that distinguish it from anteroseptal infarction. Early recognition is imperative to avoid detrimental therapies (e.g., vasodilators) and to prioritize reperfusion and preload optimization.

## Introduction

Right ventricular infarction (RVI) rarely occurs in isolation, and it accompanies inferior-posterior wall myocardial infarction (MI) in 30%–50% of patients (1). Isolated RVI occurs due to isolated occlusion of the right ventricular (RV) marginal branch or non-dominant right coronary artery (RCA) or proximal occlusion of the dominant RCA with good left coronary supply to the inferoposterior wall (2). While concomitant inferior infarction is common, isolated RVI poses unique diagnostic pitfalls due to its atypical electrocardiogram (ECG) manifestations, which may resemble anteroseptal MI (3).

A 12-lead ECG may show decremental ST-segment elevation (STE) in leads V1–V3, and STE in right-sided leads (V4R) is a hallmark of RV involvement (4, 5). Misdiagnosis carries significant morbidity, as standard therapies for left-sided infarction (e.g., nitrates and diuretics) may precipitate catastrophic hypotension in RVI (6).

This report illustrates a case of isolated RVI masquerading as anteroseptal STE infarction, emphasizing the role of systematic ECG interpretation and timely reperfusion. Accurate diagnosis is critical, as management strategies diverge significantly from those of left ventricular infarction.

## Case presentation

A 55-year-old man presented to the emergency department with chest pain lasting for 2 h. The chest pain started during heavy lifting and manifested as intense pressure below the xiphoid with profuse sweating. The patient had a history of hyperlipidemia and a long-term smoking habit.

On examination, his blood pressure was 95/58 mmHg, and his heart rate was 93 beats per minute. His oxygen saturation was 95% on room air. Laboratory tests showed that his troponin T level was 126.0 ng/L (reference range < 14.0 ng/L), creatine kinase-MB was 9.5 ng/mL (reference range < 6.2 ng/mL), and D-dimer was 0.48 mg/L (reference range <0.5 mg/L).

**Figure 1:**
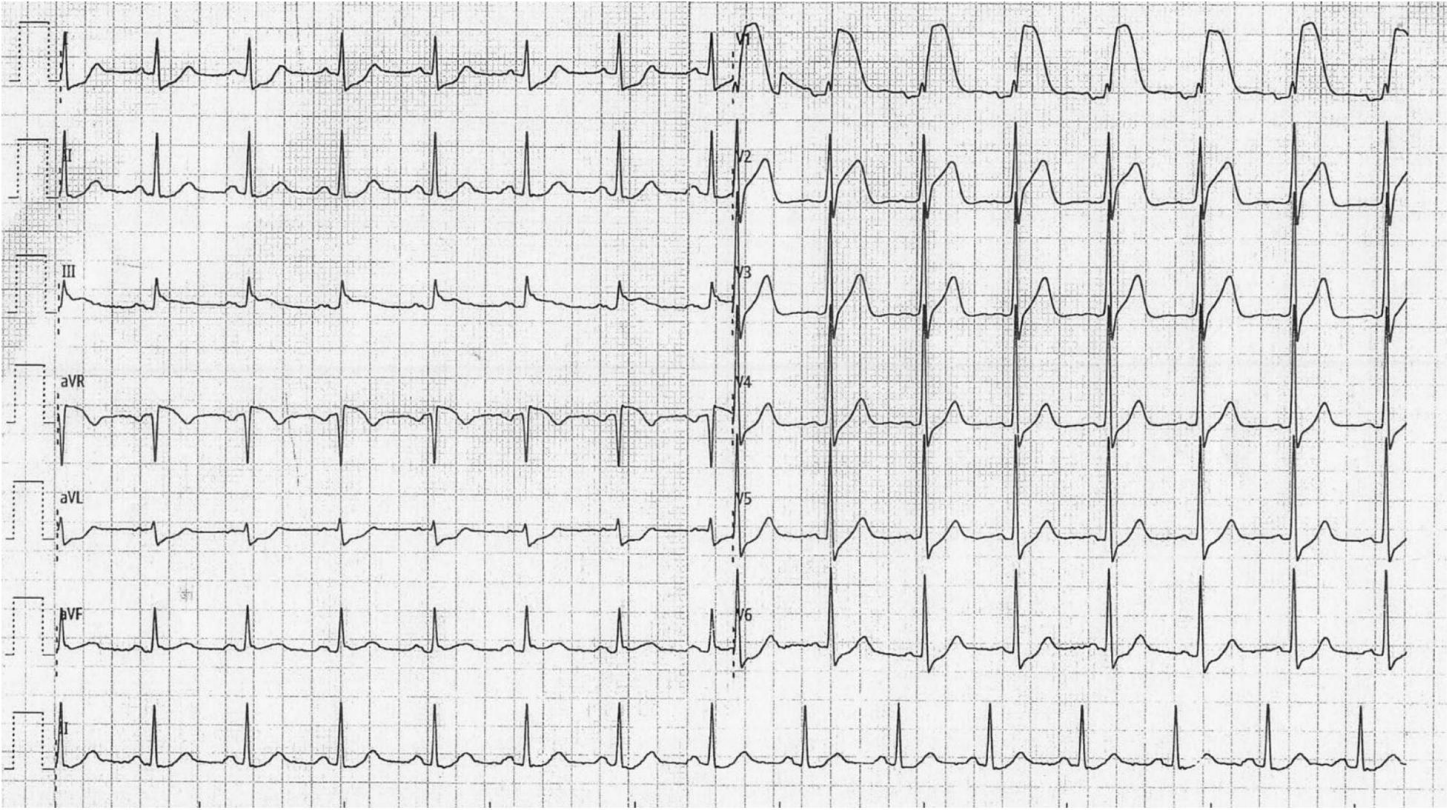
Electrocardiogram obtained on admission.

An electrocardiogram was obtained (Figure 1), showing ST-segment elevation in leads V1 and V2 with a decremental pattern (V1 > V2), concomitant ST elevation in lead III, and reciprocal ST depression in lateral leads. Right-sided ECG leads (V3R–V6R) were not recorded.

**Figure 2:**
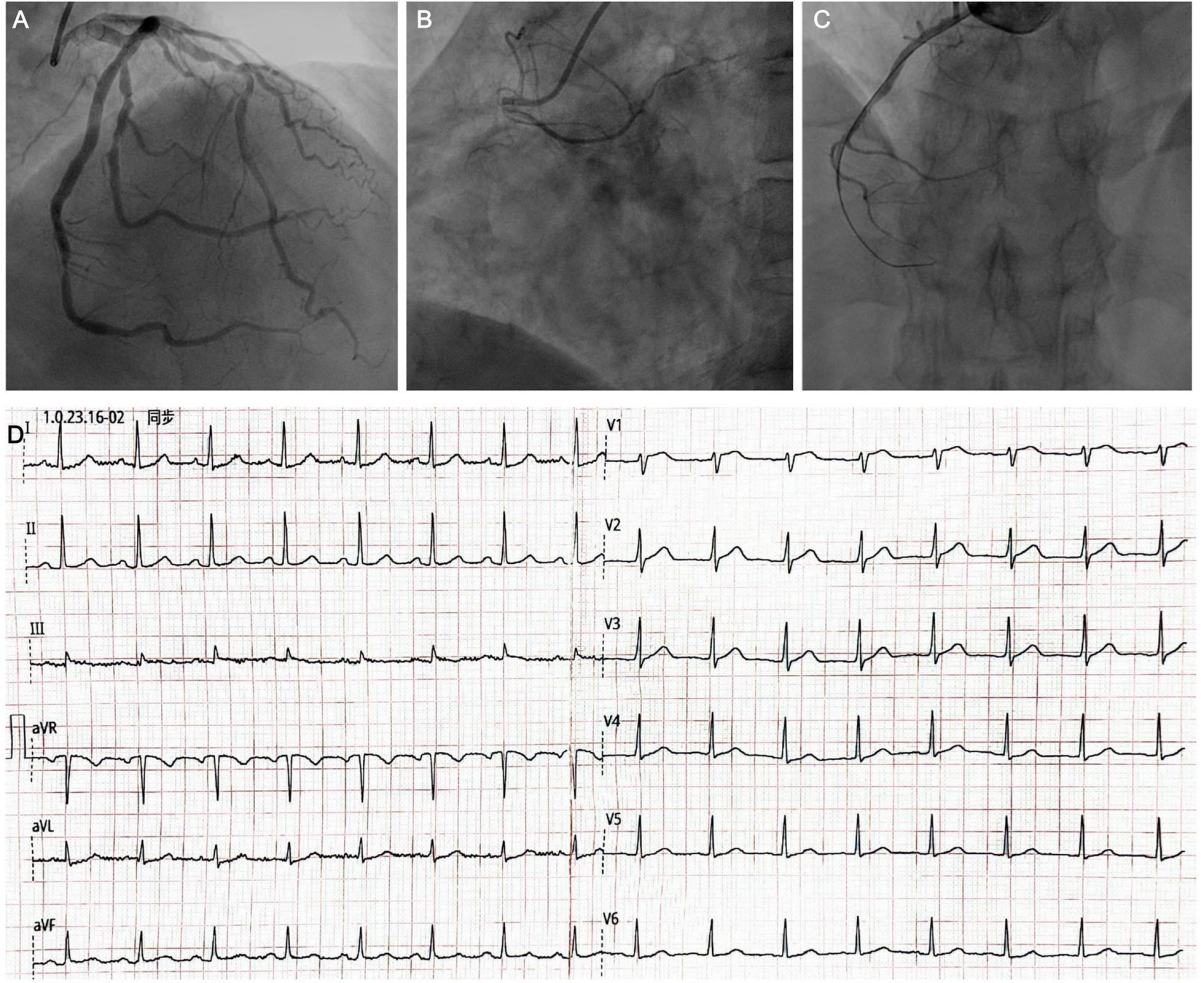
Coronary angiography and post-procedural electrocardiogram. **(A)** Coronary angiography demonstrating no significant obstruction in the left coronary artery. **(B)** Coronary angiography revealing proximal occlusion of the right coronary artery. **(C)** Coronary angiography following successful recanalization after thrombus aspiration and percutaneous coronary intervention. **(D)** Twelve-lead electrocardiogram obtained after reperfusion, showing resolution of ST-segment elevation in leads V1 and V2 and normalization of ST-segment changes in leads I and aVL.

The patient underwent emergency coronary angiography, demonstrating no significant obstruction in the left coronary artery (Figure 2A) and proximal occlusion in the RCA (Figure 2B). Thrombus aspiration was undertaken in the RCA, and reperfusion was achieved with thrombolysis in myocardial infarction grade III flow restored. The angiography confirmed that the RCA was non-dominant (Figure 2C). The electrocardiogram from the following day showed normalized ST segments in leads V1 and V2, with no Q waves in any lead (Figure 2D).

## Discussion

Typical chest pain, an elevated myocardial enzyme level, and STE in precordial leads usually suggest the diagnosis of acute anteroseptal infarction, with a lesion in the left anterior descending artery. The electrocardiogram typically presents with reciprocal ST depression in inferior leads. In contrast, the ECG of this patient displayed slightly elevated ST segments in lead III and reciprocal ST depression in lateral leads. Given the decremental STE in leads V1 and V2, isolated RVI was the most likely diagnosis.

Several key ECG features distinguish isolated RVI from anteroseptal MI (Table 1).

**Table 1:** ECG features differentiating isolated RVI from anteroseptal MI.

ECG feature	Isolated RVI	Anteroseptal MI
Precordial STE pattern	Decremental (V1 > V2 > V3)	Incremental (V1 < V2 < V3)
Maximal STE location	V1	V2–V3
Inferior leads (II, III, and aVF)	STE, especially lead III	ST depression (reciprocal)
Lateral leads (I, aVL, and V5–V6)	ST depression (reciprocal)	No significant change
Right-sided leads (V4R)	STE ≥ 0.1 mV	No STE
Associated findings	Hypotension	Variable

RVI is usually accompanied by inferior wall infarction due to occlusion of the dominant RCA, characterized by prominent STE in inferior leads. Isolated acute RVI is less commonly encountered and is frequently misdiagnosed as anterior wall infarction when precordial STE occurs exclusively. Differential diagnosis is essential as adequate preload is needed in RVI. Drugs routinely used in left ventricular infarction, such as vasodilators and diuretics, may reduce cardiac output and cause severe hypotension (2). As a consequence, vasodilators should be avoided, and early reperfusion is imperative.

On a 12-lead electrocardiogram, decremental STE in leads V1 to V3 (possibly to V5) suggests RVI, while anterior wall infarction displays incremental STE (3). In cases with comparable STE amplitude, STE ≥ 0.1 mV in the right precordial leads (especially V4R) is highly sensitive and specific for identifying RVI (3). STE in lead III or reciprocal ST depression in leads I and aVL may also be detected, which are sensitive but not specific for RVI (7).

## Practical diagnostic algorithm in emergency settings

When encountering a patient with chest pain and precordial STE in leads V1–V3:

Step 1: Assess the ST elevation pattern

If decremental (V1 > V2) → Consider RVI

Step 2: Check inferior leads

If STE in lead III **→** Supports RVI

If ST depression in leads II, III, and aVF **→** Suggests anteroseptal MI

Step 3: Evaluate lateral leads

If reciprocal ST depression in leads I and aVL **→** Supports RVI

Step 4: Obtain right-sided ECG (V3R–V6R)

If STE ≥ 0.1 mV in V4R → Confirms RVI

Step 5: Assess hemodynamics

If hypotension/low cardiac output **→** Suspect RVI

## Limitations

This case report has several limitations inherent to single-case studies. First, the generalizability of findings is limited, and larger case series would be needed to validate the diagnostic approach. Second, right-sided ECG leads (V3R–V6R) were not obtained in this case, which would have provided additional diagnostic confirmation. Third, echocardiographic assessment of RV function was not performed immediately, which could have provided valuable hemodynamic information. Despite these limitations, this case effectively illustrates the diagnostic challenge and key ECG features of isolated RVI.

## Highlights

Isolated RVI may mimic anteroseptal MI but requires distinct management.
Decremental precordial STE (V1 > V2), concomitant STE in lead III, and reciprocal lateral ST depression are key distinguishing features.
Right-sided ECG leads should be obtained when RVI is suspected.
Vasodilators and diuretics must be avoided in RVI.
Early reperfusion and preload optimization are cornerstones of management.
Maintaining adequate RV preload is critical to prevent hemodynamic collapse.

This case illustrates that isolated RVI due to occlusion of the non-dominant RCA is a diagnostic challenge that can be confused with anteroseptal MI. The key distinguishing feature is the decremental ST-elevation pattern in precordial leads combined with concomitant inferior lead elevation and reciprocal lateral ST depression. Early recognition through systematic ECG analysis is crucial to avoid the potentially catastrophic administration of vasodilators and to implement appropriate management focused on preload optimization and urgent reperfusion. Clinicians should maintain a high index of suspicion for isolated RVI in patients presenting with precordial ST elevation, particularly when atypical features are present.

## Data Availability

The original contributions presented in the study are included in the article/Supplementary Material, further inquiries can be directed to the corresponding author.

## References

[1] Haji SA Movahed A. Right ventricular infarction–diagnosis and treatment. Clin Cardiol. (2000) 23(7):473–82. 10.1002/clc.4960230721 10894433 PMC6654868

[2] Kinch JW Ryan TJ. Right ventricular infarction. N Engl J Med. (1994) 330(17):1211–7. 10.1056/NEJM199404283301707 8139631

[3] Iannetta L Puddu PE Missiroli B Morabito G Grillo P De Gregorio C Pathophysiology and ECG patterns of isolated right ventricular infarction with nondominant right coronary artery. J Cardiovasc Med. (2013) 14(10):740–4. 10.2459/JCM.0b013e32835853a3 22914309

[4] Wellens HJJ Conover MB. The ECG in Emergency Decision Making. 2nd ed. Philadelphia, PA: Saunders (2006).

[5] Braat SH Gorgels AP Bar FW Wellens HJ. Value of the ST-T segment in lead V4R in inferior wall acute myocardial infarction to predict the site of coronary arterial occlusion. Am J Cardiol. (1988) 62(1):140–2. 10.1016/0002-9149(88)91380-x 3289355

[6] Dell’Italia LJ Starling MR Blumhardt R Lasher JC O'Rourke RA. Comparative effects of volume loading, dobutamine, and nitroprusside in patients with predominant right ventricular infarction. Circulation. (1985) 72(6):1327–35. 10.1161/01.cir.72.6.1327 4064277

[7] Verouden NJ Barwari K Koch KT Henriques JP Baan J van der Schaaf RJ. Distinguishing the right coronary artery from the left circumflex coronary artery as the infarct-related artery in patients undergoing primary percutaneous coronary intervention for acute inferior myocardial infarction. Europace. (2009) 11(11):1517–21. 10.1093/europace/eup234 19706635

